# Supplementary figures and images for: A molecular phylogeny of the Chinese Sinopoda spiders (Sparassidae, Heteropodinae): implications for taxonomy
Source: PeerJ. 2021 Aug 20;9:e11775. doi: 10.7717/peerj.11775 (PMC8381878; doi:10.7717/peerj.11775)

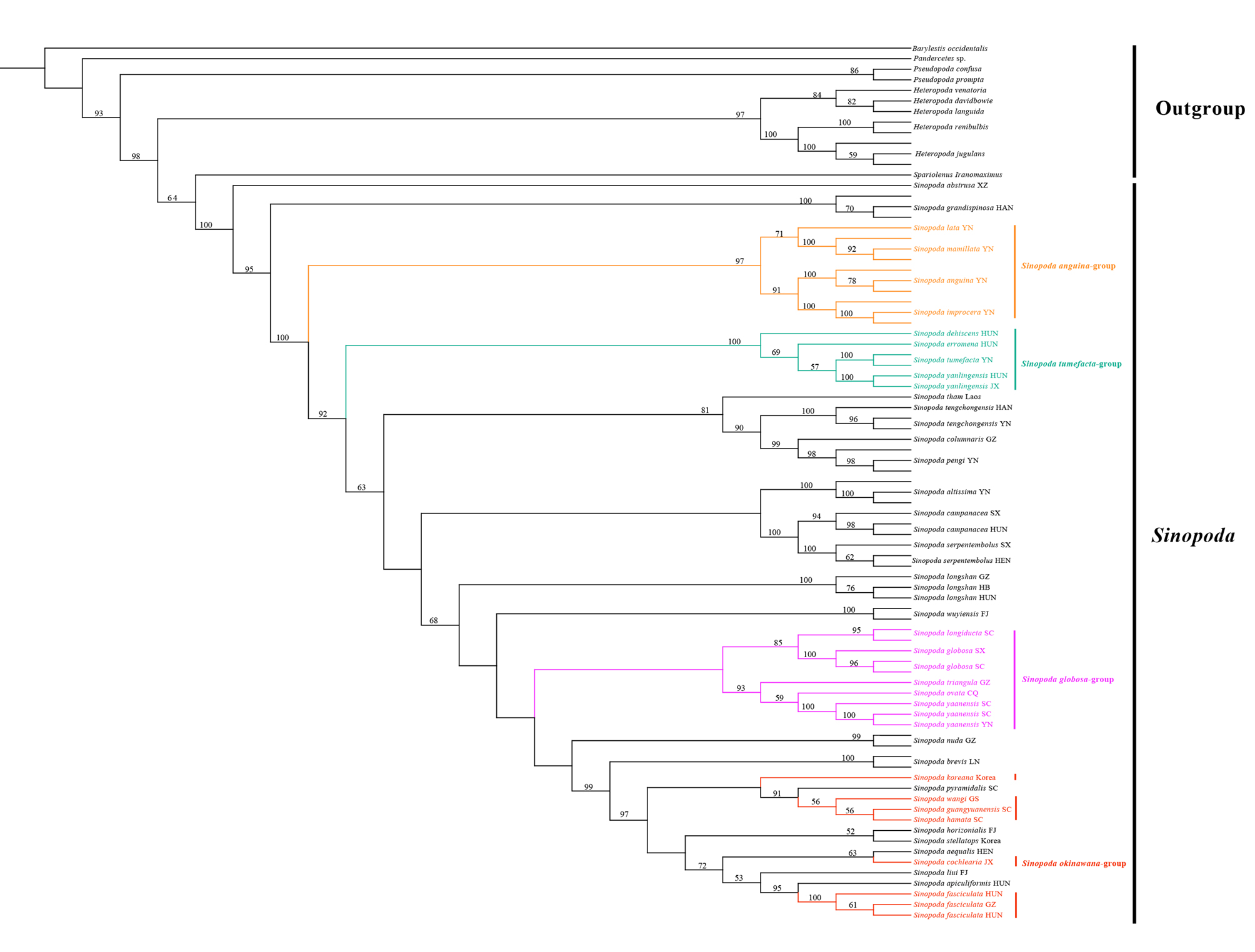

Supplement: Supplemental Information 3 — The numbers at the nodes represent bootstrap support values from likelihood analyses. [file peerj-09-11775-s003.jpg]
